# Effect of Hypoxia Conditioning on Body Composition in Middle-Aged and Older Adults: A Systematic Review and Meta-Analysis

**DOI:** 10.1186/s40798-023-00635-y

**Published:** 2023-09-25

**Authors:** Zhijian He, Lijun Qiang, Yusheng Liu, Wenfeng Gao, Tao Feng, Yang Li, Bing Yan, Olivier Girard

**Affiliations:** 1 China Institute of Sport and Health Science, Beijing Sport University, No. 48 Xinxi Road, Haidian District, Beijing, 100084 China; 2 Department of Sports Teaching and Research, Lanzhou University, Lanzhou, China; 3 Ningxia Vocational College of Sports, Ningxia, China; 4 Tsinghua University High School (Guanghua), Beijing, China; 5 School of Human Sciences (Exercise and Sport Science), The University of Western Australia, Perth, WA Australia

**Keywords:** Hypoxic training, Normobaric hypoxia, Body fat, Lean mass, Older adults

## Abstract

**Background:**

The effects of hypoxia conditioning, which involves recurrent exposure to hypoxia combined with exercise training, on improving body composition in the ageing population have not been extensively investigated.

**Objective:**

This meta-analysis aimed to determine if hypoxia conditioning, compared to similar training near sea level, maximizes body composition benefits in middle-aged and older adults.

**Methods:**

A literature search of PubMed, EMBASE, Web of Science, Scopus and CNKI (China National Knowledge Infrastructure) databases (up to 27^th^ November 2022) was performed, including the reference lists of relevant papers. Three independent reviewers extracted study characteristics and health outcome measures. Search results were limited to original studies of the effects of hypoxia conditioning on body composition in middle-aged and older adults.

**Results:**

Twelve studies with a total of 335 participants were included. Hypoxia conditioning induced greater reductions in body mass index (MD = -0.92, 95%CI: -1.28 to -0.55, I^2^ = 0%, p < 0.00001) and body fat (SMD = -0.38, 95%CI: -0.68 to -0.07, I^2^ = 49%, p = 0.01) in middle-aged and older adults compared with normoxic conditioning. Hypoxia conditioning improved lean mass with this effect not being larger than equivalent normoxic interventions in either middle-aged or older adults (SMD = 0.07, 95%CI -0.12 to 0.25, I^2^ = 0%, p = 0.48). Subgroup analysis showed that exercise in moderate hypoxia (FiO_2_ > 15%) had larger effects than more severe hypoxia (FiO_2_ ≤ 15%) for improving body mass index in middle-aged and older adults. Hypoxia exposure of at least 60 min per session resulted in larger benefits for both body mass index and body fat.

**Conclusion:**

Hypoxia conditioning, compared to equivalent training in normoxia, induced greater body fat and body mass index improvements in middle-aged and older adults. Adding hypoxia exposure to exercise interventions is a viable therapeutic solution to effectively manage body composition in ageing population.

## Key Points

- A meta-analysis was conducted to evaluate the effects of hypoxia conditioning on body composition in middle-aged and older adults.
- Compared to normoxic equivalent, hypoxia conditioning provides greater reductions in body fat and body mass index. However, the magnitude of improvement in lean mass was not greater in hypoxic than normoxic conditions.
- Hypoxia conditioning of at least 60 minutes per session in moderate hypoxia (FiO_2_ > 15%) is a promising intervention to improve body composition in middle-aged and older adults.

## Background

The number and proportion of middle-aged adults (ages 40–65 years) and older adults (aged older than 65 years) is increasing globally [1]. Body composition changes occur with ageing, including fat accumulation and muscle loss, which may lead to obesity and sarcopenia in ageing population [2–4]. A smaller muscle mass is related to cardiovascular and metabolic diseases, compromised quality of life, increased risk of falls, fractures, and mortality [5–8]. Obesity is another important health risk factor affecting middle-aged and older adults that leads to insulin resistance, chronic inflammation, cardiovascular diseases, frailty, and mental disorders [9–11]. Therefore, identifying effective, enjoyable, and safe interventions to improve body composition in ageing population is a priority.

Exercise is a cornerstone therapy to improve body composition by reducing body fat and signalling pathways controlling muscle mass [12–14]. Various exercise modalities including aerobic training, high-intensity interval training and resistance training can improve body composition in the elderly [15, 16]. Compared to those without training, older adults with sarcopenia obesity undertaking eight weeks of moderate-intensity aerobic training, resistance training, and combined aerobic and resistance training interventions significantly improved muscle mass and fat mass [17]. Twelve weeks of elastic-band resistance training combined with walking/running on a treadmill and bicycle significantly decreased body weight, body mass index (BMI), and body fat percentage in obese, older men [18]. While exercise training provides valuable health benefits for managing and even reversing some signs of ageing, no single exercise modality can be recommended.

Exercising regularly, at a moderate-to-vigorous intensity, and combining activities that challenge the heart, lungs and muscles, assists in managing cardio-metabolic health [19–21]. Higher exercise intensities have generally been associated with larger improvement in body composition [22–25]. For instance, training at an intensity corresponding to 70% compared to 50% of maximal oxygen consumption leads to larger benefits for fat mass, total abdominal fat, and subcutaneous abdominal fat in middle-aged females [22]. Despite larger improvement in body composition, exercising at a higher intensity is eventually accompanied by higher injury risk [26] and reduced pleasure [27], likely preventing its wide application in sedentary populations. Furthermore, sedentary middle-aged and older adults are often suffering from an increased prevalence of chronic musculoskeletal disorders (i.e., osteoarthritis or osteoporosis), in turn decreasing training adherence especially for higher intensity workouts [28]. Innovative approaches are warranted for effective body composition management in ageing populations beyond what is achieved to date.

Hypoxia conditioning relates to active (i.e., during exercise) exposure to systemic (whole body) and/or local (tissue) hypoxia, resulting in a decrease in arterial oxygen availability [29]. During the last decade, the popularity of training at low-to-moderate exercise intensity combined with systemic hypoxia (low oxygen conditions) to bring equivalent or additional physiological and functional benefits, compared with high-intensity exercise in normoxia (normal oxygen conditions), has grown in obese cohorts [30]. Several studies recruiting young, apparently healthy individuals have demonstrated that resistance training in hypoxia is effective for improving skeletal muscle fibre cross-sectional area, lean body mass, muscle strength, and exercise capacity [31–36]. To date, however, whether hypoxia conditioning compared to normoxia leads to improved body composition in middle-aged and older adults remains controversial. Training in hypoxia (inspired oxygen fraction [FiO_2_] = 15%) twice weekly for four weeks induced larger improvements in physical fitness and body composition than similar training in normoxia, with also the advantage of lower sustained workloads during actual training sessions in hypoxia [37]. In overweight and obese adults, training in hypoxia was also more effective at reducing abdominal fat than in normoxia [38, 39]. Conversely, others failed to report greater improvements in markers of body composition (i.e., BMI, total lean mass, and total fat mass) after training in oxygen-deprived conditions [35, 40–43].

Therefore, this meta-analysis aimed to determine if hypoxia conditioning, compared to similar training near sea level, maximizes body composition benefits in middle-aged and older adults.

## Methods

This meta-analysis was conducted in accordance with the Preferred Reporting Items for Systematic Review and Meta-analyses (PRISMA) 2020 Statement [44].

### Search strategy

A literature search was conducted using PubMed, EMBASE, Web of Science, Scopus and CNKI (China National Knowledge Infrastructure) databases. In addition, references of the included studies and previously published reviews were manually searched. Studies were searched in the electronic databases using the following key terms combined by Boolean logic (“AND”, “OR”, “NOT”): ‘hypoxia’, ‘altitude’, ‘normobaric’, ‘hypoxic exposure’, ‘exercise’, ‘training’, ‘high-intensity interval training’, ‘resistance exercise’, ‘aerobic exercise’, ‘strength training’, ‘weight training’, ‘body composition’, ‘body fat’, ‘lean mass’, ‘body mass index’, ‘muscle mass’, ‘fat-free mass’, ‘middle-aged’, ‘aged’, ‘older adult’, ‘elderly’, ‘old people’, ‘postmenopausal women’. The search of titles, abstracts and key words was conducted on 27^th^ November 2022.

### Eligibility criteria

Each eligible study had to meet all of the following criteria for the type of study, participants, interventions, and outcomes: (1) randomized controlled trials; (2) participants aged 40 years and older with no serious diseases; (3) the study design included comparisons between the same exercise training protocol in hypoxia (exposures were required to be simulated altitude ≥ 2000 m or a FiO_2_ < 16.4%) and normoxia condition; (4) reported outcomes of body composition (BMI, lean body mass, body fat percentage, fat-free mass and/or fat mass). Studies were excluded if at least one of the following criteria were met: (1) abstracts or reviews; (2) not written in English or Chinese; (3) non-randomized controlled trials; (4) participants aged under 40; (5) participants with severe diseases including cancer, cardiovascular (i.e., heart failure and coronary artery disease), neurological (i.e., Parkinson's disease, Alzheimer's disease, multiple sclerosis), and respiratory (i.e., end-stage chronic obstructive pulmonary disease, pulmonary fibrosis, or severe asthma) conditions; (6) blood flow restriction as the hypoxia intervention; (7) full-text or data extraction unavailable; (8) no physical activity or exercise prescribed as part of the intervention protocol. Titles and abstracts of the initial retrieved studies were assessed by three independent researchers, any disagreement was discussed until reaching consensus. Subsequently, the full text of the potentially eligible studies was independently evaluated by two researchers. Disagreements were solved initially via discussion between the two independent researchers, while a third researcher was consulted for dispute resolution.

### Data extraction

Data were extracted independently by two researchers into a pre-designed spreadsheet, including: (1) basic information (i.e., authors, year of publication); (2) experimental design (i.e., frequency, duration, intervention); (3) participant characteristics (i.e., age, sex, BMI and the number of participants); (4) outcome data suitable for analysis based on mean, standard deviation (SD) and sample size. The corresponding author of the included study would be contacted directly if the original data were not reported or incomplete. For studies that did not report outcomes including pre-post change as “Mean ± SD” or the outcomes were presented as “Mean ± SE/SEM (standard error/ standard error of mean)”, calculations were conducted using the following formulas:

$$\begin{aligned} & Mean = Mean_{2} - Mean_{1} ; SD = \sqrt[2]{{SD_{1}^{2} + SD_{2}^{2} - 2SD_{1}^{2} \times SD_{2}^{2} \times R}} \\ & SD_{1} = SE_{1} \times \sqrt n ;SD_{2} = SE_{2} \times \sqrt n \\ \end{aligned}$$

*SD*_1_, *SD*_2_ = standard deviation of pre and post; *SE*_1_, *SE*_2_ = standard error of pre and post; *Mean*_2_, *Mean*_1_ = Mean of pre and post; R = 0.4/0.5

Values presented as figures were digitized using graph digitizer software (WebPlotDigitizer), and the means and SD were measured manually at the pixel level to the scale provided on the figure.

### Methodological quality assessment

The quality of each included study was determined by two researchers independently using The Cochrane Collaboration's tool for assessing risk of bias [45], followed by a cross-check of the results. The assessment was conducted according to the Cochrane manual 5.1.0. from the following domains: (1) random sequence generation; (2) allocation concealment; (3) blinding of participants, personnel, and outcome assessors; (4) blinding of outcome assessment; (5) incomplete outcome data; (6) selective reporting; (7) other sources of bias. A judgement was made on each of the domains as to whether studies were ‘low risk’, ‘high risk’ or ‘unclear risk’. Any disagreement between the two researchers on assessment results was solved initially via discussion, while a third researcher was consulted for dispute resolution.

### Statistical analysis

All statistical analyses were conducted using the Review Manager software (version 5.3, the Cochrane Collaboration, Oxford, UK). Heterogeneity between trials was assessed using the I^2^ statistic and the *Chi-squared* test. The Cochrane guidelines explain I^2^ statistics as follows: I^2^ < 25%, *low* heterogeneity is assumed; I^2^ < 75% and > 25%, *moderate* heterogeneity is assumed; I^2^ > 75%, *high* heterogeneity is assumed [46]. The random-effects model was chosen when I^2^ > 25%, otherwise the fixed-effects model was used. If the outcomes were continuous variables, the sample size, the mean values, and SDs were extracted and used for statistical analyses. Besides, mean difference (MD) with 95% confidence intervals (95%CI) or standardized mean difference (SMD) with 95%CI were used based on whether the outcome data were reported in the same unit or not [45]. The significance was set at p < 0.05. The criteria to interpret the magnitude of the effect size (Cohen’s *d* effect size) were as follows: < 0.2 = *no* effect, 0.20–0.49 = *small* effect, 0.50–0.79 = *moderate* effect, and ≥ 0.80 = *large* effect. Small study effects were explored using funnel plots of MD *versus* SE, and by quantifying Egger’s linear regression intercept [45]. A large and statistically significant Egger statistic indicates the presence of a small study effect. Subgroup analyses were performed for investigating potential moderators including age (middle-aged and older adults), hypoxia severity (FiO_2_ = 15% as the cut off point for moderate hypoxia [47]) and hypoxia exposure duration per session (< 60 min and ≥ 60 min).

## Results

### Search results

**Fig. 1:**
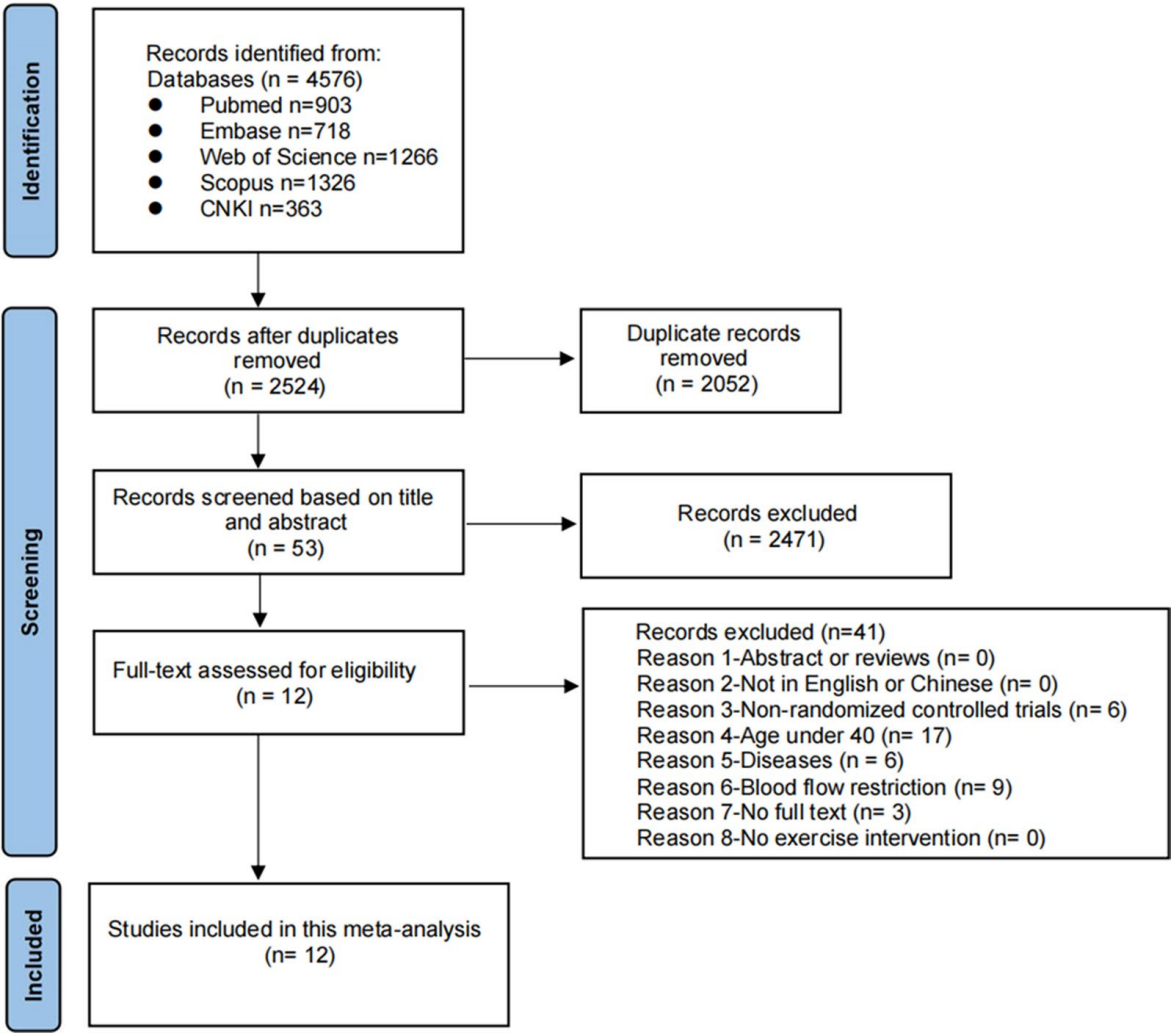
Flow diagram of study selection

A total of 4576 studies were retrieved from database screening (PubMed: n = 903, Embase: n = 718, Web of Science: n = 1266, Scopus: n = 1326, CNKI: n = 363). Initially, 2052 duplicate studies were identified and then removed using the EndNote reference management software (version X9, Clarivate Analytics). Subsequently, 2471 studies were excluded according to the titles and abstracts, and 53 studies were identified as suitable for further assessment. Following the full screening process, 12 studies (Table 1) were included in this meta-analysis [35, 37, 40–43, 48–53] (Fig. 1).

**Table 1:** Studies investigating changes in body composition markers following exercise training in hypoxia compared to normoxia in middle-aged and older adults

Study	Participant Characteristics				Intervention							
	Participants (M/F)		Age		BMI	Duration	Frequency	Type	Exercise intensity	Hypoxia severity/duration per session	Type of hypoxia	Outcome
	HYP	NOR	HYP	NOR								
Abdallah et al. [52]	16 (10/6)	15 (13/2)	51.0 ± 8.3	52.0 ± 7.5	H:31.5 ± 4.0 N:32.4 ± 4.8	8wks	3days/wk	High-intensity interval training	80% W_peak_ (HYP)/ 100% W_peak_ (NOR)	FiO_2_ = ~ 12%/35-48min	Normobaric	BMI, LM, FM
Allsopp et al. [42]	10 (6/4)	10 (6/4)	65.9 ± 1.1	64.0 ± 0.8	H: 24.9 ± 1.1 N: 23.9 ± 0.8	8wks	2days/wk	Resistance	70% 1RM	FiO_2_ = 14.4%/60min	Normobaric	BMI, LM, FM (total, leg, arm, trunk)
Camacho et al. [49]	10 (3/7)	11 (6/5)	73.5 ± 4.7	70.2 ± 6.4	H: 28.9 ± 4.2 N: 29.5 ± 4.8	18wks	2days/wk	Whole-body vibration	2.54 G12.6 Hz/4 mm	FiO_2_ = 16.2%/16min	Normobaric	LM% (total, trunk, right leg)
Camacho et al. [50]	9 (2/7)	10 (5/5)	72.6 ± 3.6	68.8 ± 5.3	H: 28.3 ± 4.4 N: 27.8 ± 5.8	18wks	2days/wk	Whole-body vibration	12.6 Hz/4 mm	FiO_2_ = 16.2%/21min	Normobaric	LM
Chacaroun et al. [43]	12 (11/1)	11 (8/3)	52.0 ± 12.0	56.0 ± 11.0	H: 31.2 ± 2.4 N: 31.8 ± 3.2	8wks	3days/wk	Aerobic	75% HR_max_	FiO_2_ = ~ 13.0%/45min	Normobaric	BMI, LM/ LM%, FM/ FM%
Hanneset al. [53]	16 (4/12)	16 (6/10)	50.3 ± 10.3	52.4 ± 7.9	H: 37.9 ± 8.1 N: 36.3 ± 4.0	8months	2days/wk	Aerobic	65–70% HR_max_	FiO_2_ = ~ 14.0%/180min	Normobaric	BMI, MM%, FM%
Nishiwaki et al. [48]	7 (0/7)	7 (0/7)	56.0 ± 1.0	56.0 ± 1.0	> 24.0	8wks	4days/wk	Aerobic	50% V̇O_2peak_	Simulation of 2000 m (~ 600 mmHg)/ 120min	Hypobaric	BMI, LM, BF (subcutaneous, preperitoneal, total%)
Park et al. [51]	12 (12/0)	12 (12/0)	66.5 ± 0.9	66.5 ± 0.7	H: 26.0 ± 0.6 N: 25.6 ± 0.4	12wks	3days/wk	Aerobic + Resistance	60%-70% HR_max_/60%-70% 1RM	FiO_2_ = 15.0%/90-120min	Normobaric	LM, BF%
Pramsohler et al. [41]	19 (7/12)	16 (5/11)	80.2 ± 7.2	82.0 ± 7.8	H: 25.2 ± 5.3 N: 25.8 ± 5.7	3wks	7days/3wks	Aerobic	80% eV̇O_2peak_	FiO_2_ = 15.3%/30min	Normobaric	BMI
Timon et al. [40]	17 (NR)	18 (NR)	68.5 ± 3.8	70.4 ± 3.3	H: 26.4 ± 3.3 N: 27.1 ± 3.9	24wks	3days/wk	Resistance	RPE 6–8	FiO_2_ = 16.1%/45min	Normobaric	LM, FM
Törpel et al. [35]	19 (9/10)	17 (9/8)	68.1 ± 4.6	67.8 ± 4.1	H: 27.6 ± 4.2 N: 26.9 ± 3.6	5wks	4days/wk	Resistance	40% 1RM	SpO_2_ = ~ 80–85%/180min	Normobaric	LM, FM
Wiesner et al. [37]	24 (10/14)	21 (8/13)	42.2 ± 1.2	42.1 ± 1.7	H: 33.1 ± 0.3 N: 32.5 ± 0.8	4wks	3days/wk	Aerobic	65% V̇O_2max_	FiO_2_ = 15%/60min	Normobaric	BMI, LM%, FM%

*M* male, *F* female, *NR* not reported, *HYP* hypoxia, *NOR* normoxia *BMI* body mass index, *wks* weeks, *RM* repetition maximum, *RPE* rating of perceived exertion, *mins* minutes, *FiO*_*2*_ fraction of inspired oxygen, *SpO*_*2*_ arterial oxygen saturation, *W*_*peak*_ maximal workload, *V̇O*_*2peak*_ peak oxygen uptake, *V̇O*_*2max*_ maximal oxygen uptake, *HR*_*max*_ maximal heart rate, *LM* lean mass, *FM* fat mass, *FM%* fat mass percentage, *LM%* lean mass percentage, *MM%* muscle mass percentage, *BF* body fat

### Study characteristics

A total of 335 participants (152 men, 148 women, and 35 participants with unreported sex) aged 40 to 80 were included. Hypoxia severity ranged from ~ 2000 m to 4500 m simulated altitudes or FiO_2_ = 16.4–12.0% (mean: ~ 2264 m or FiO_2_ = 12.5%). Duration of hypoxic exposure ranged from 16 to 180 min (Mean: ~ 77 min). Studies implemented resistance exercise (n = 3), aerobic exercise (n = 5), whole-body vibration training (n = 2), high-intensity interval training (n = 1), and aerobic combined with resistance exercise (n = 1). Lean mass was reported in eleven studies, while BMI and body fat were reported in seven and nine studies, respectively (Table 1).

### Risk of bias of included studies

**Fig. 2:**
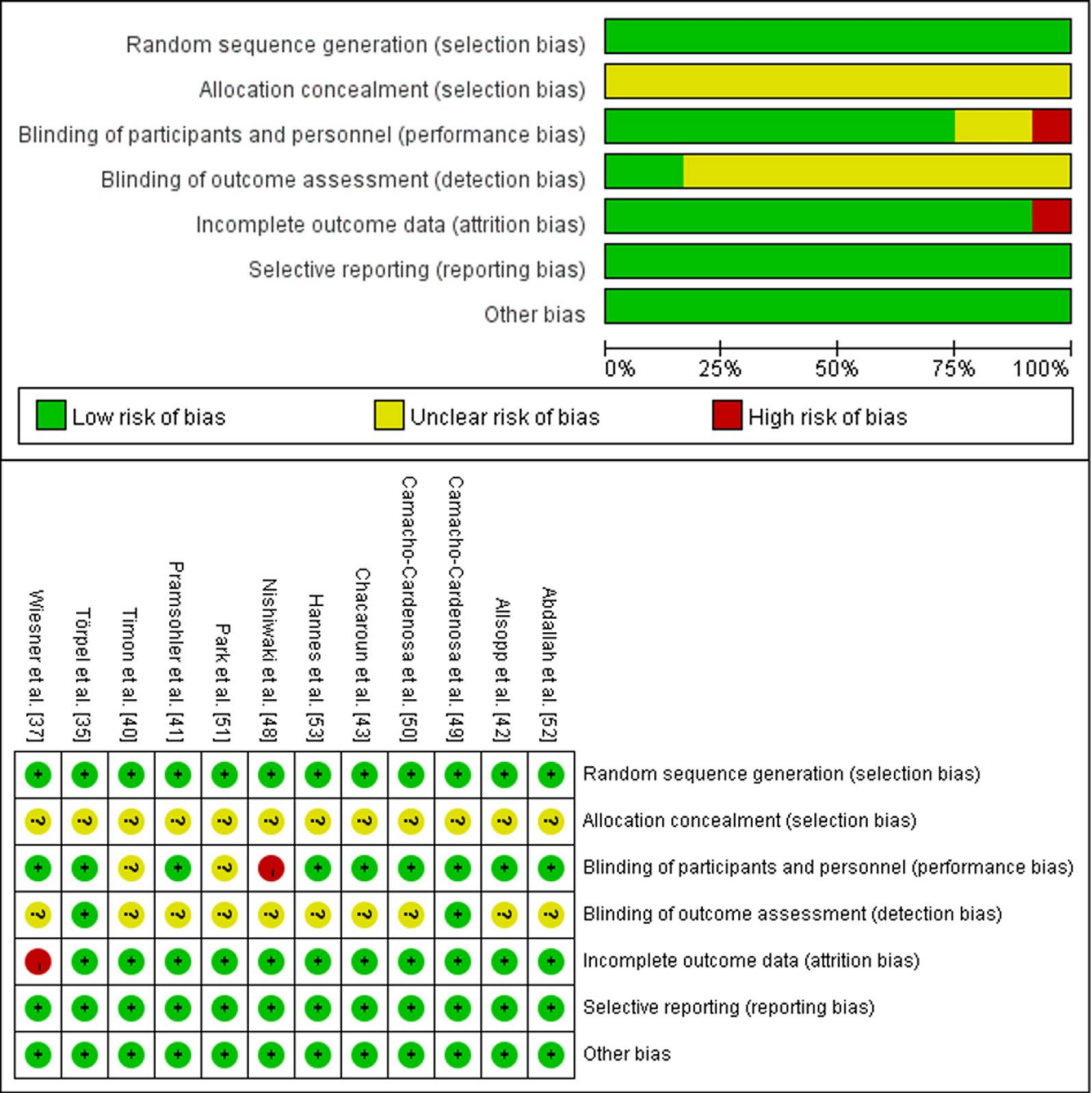
Risk of bias summary of included studies

All included studies used randomization methods, while none of them reported the specific methods of allocation concealment (evaluated as ‘unclear risk’ of bias). The blinding of participants and personnel was not described clearly in two studies and was evaluated as a ‘high risk’ of bias in one study. Besides, ten studies did not report the blinding of outcome assessment (‘unclear risk’ of bias), while two studies were classified as ‘low risk’ of bias. Except for one study that was considered as ‘high risk’ of bias for incomplete outcome data, all the other studies were evaluated as ‘low risk’ of bias for the three assessment domains (incomplete outcome data, selective reporting, and other bias). Given that the use of complete blinding methods may not be feasible in exercise interventions, the overall risk of bias in the included studies was evaluated as ‘low-to-moderate’ (Fig. 2).

### Results of quantitative synthesis

#### Lean mass

**Fig. 3:**
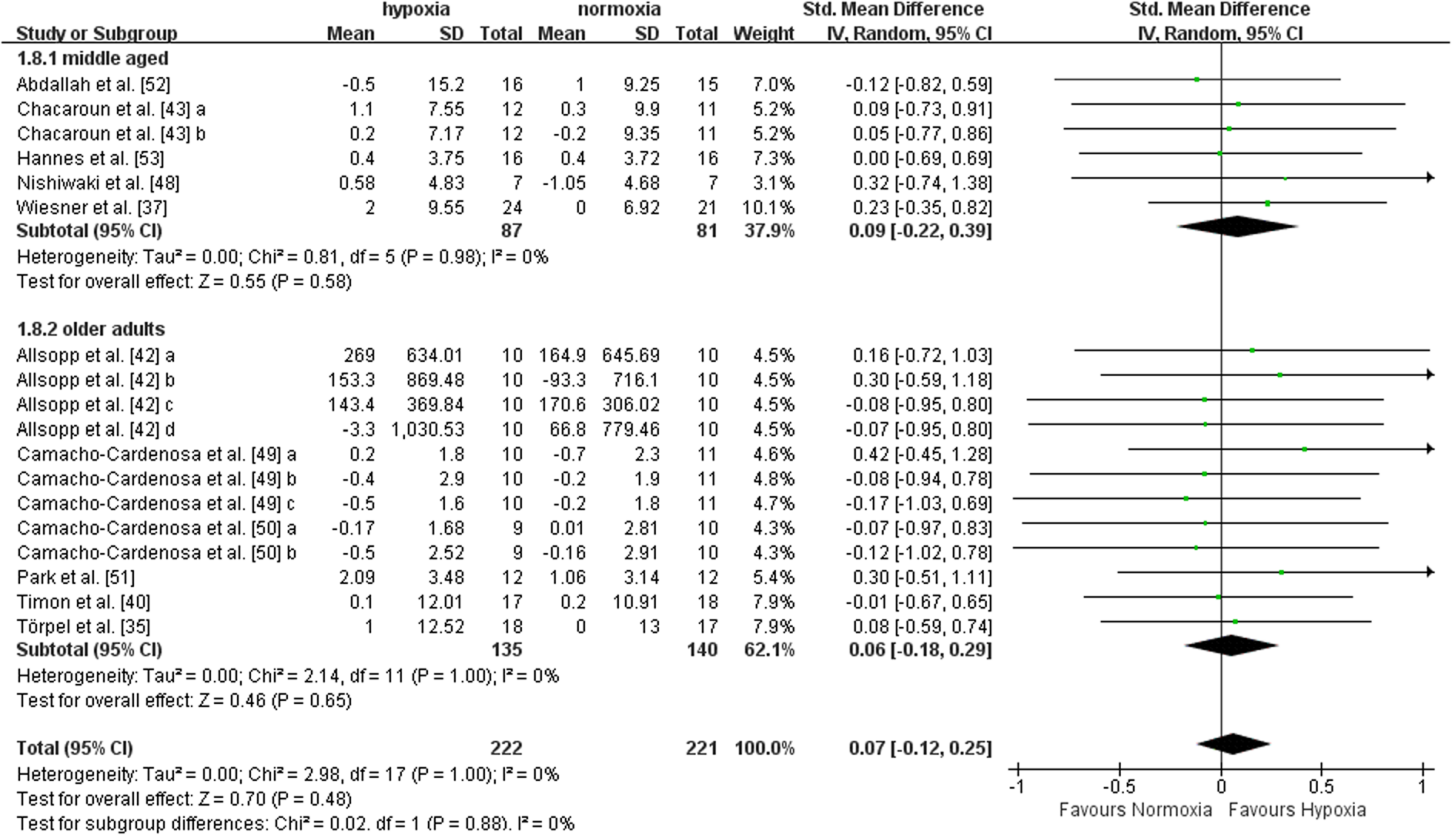
Meta-analysis of the effects of exercise in hypoxia *versus* normoxia on lean mass in middle-aged and older adults. ‘a’, ‘b’, ‘c’, ‘d’ represents different outcome indicators of lean mass reported in the same study. Filled green square represents study-specific estimates, and filled diamond represents pooled estimates of random-effects. *CI* confidence interval, *SD* standard deviation

Among the twelve selected studies, eleven studies reported outcome indicators concerning lean mass (i.e., fat-free mass, lean body mass, trunk, and limb lean mass, and muscle mass). Five studies included middle-aged adults, and six recruited older adults. A total of 18 data points were included in the quantitative synthesis. The effect of exercise in hypoxia *versus* normoxia on lean mass was not statistically different (SMD = 0.07, 95%CI -0.12 to 0.25, I^2^ = 0%, p = 0.48). The effects were not different between middle-aged (SMD = 0.09, 95%CI -0.22 to 0.39, I^2^ = 0%, p = 0.58) and older adults (SMD = 0.06, 95%CI -0.18 to 0.29, I^2^ = 0%, p = 0.65), according to subgroup analysis (Fig. 3).

#### BMI

**Fig. 4:**
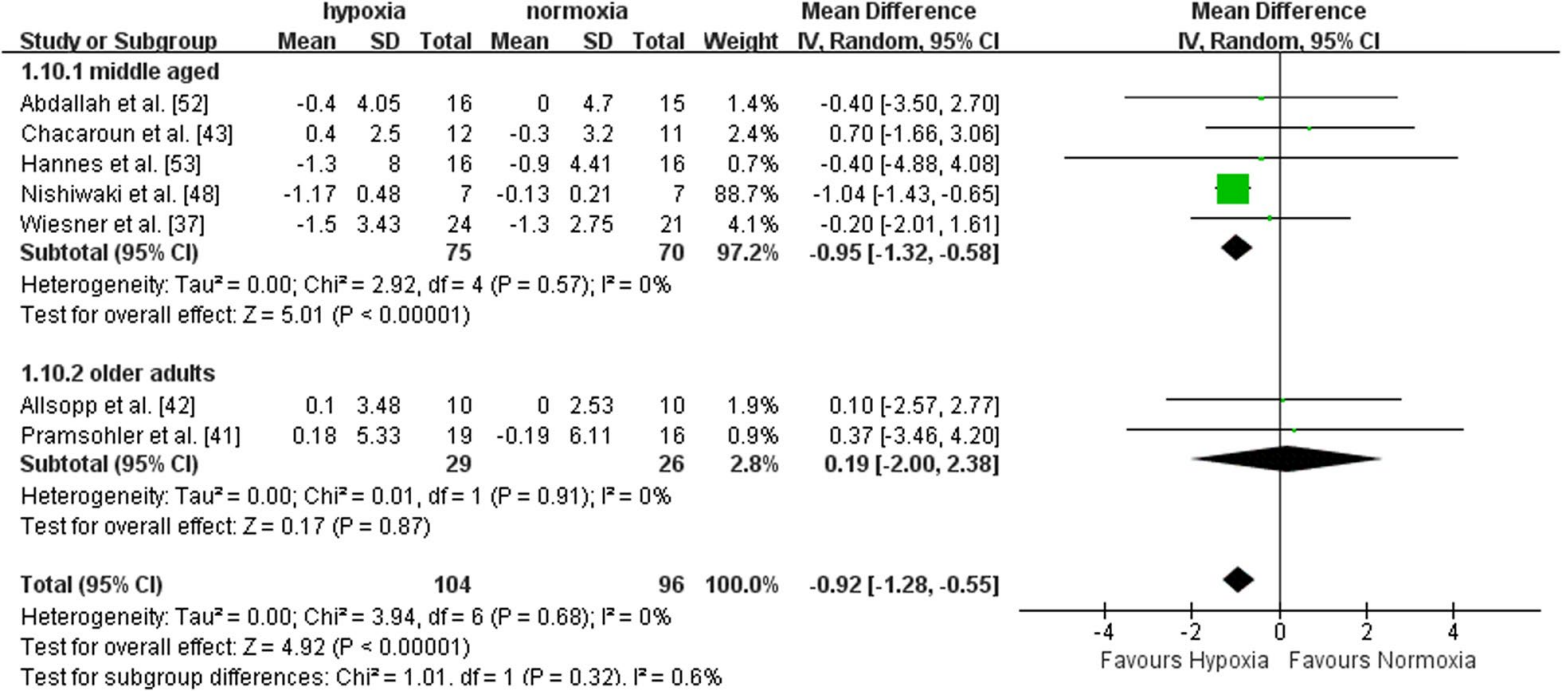
Meta-analysis of the effects of exercise in hypoxia *versus* normoxia on BMI in middle-aged and older adults. Filled green square represents study-specific estimates, and filled diamond represents pooled estimates of random-effects. *CI* confidence interval, *SD* standard deviation

**Fig. 5:**
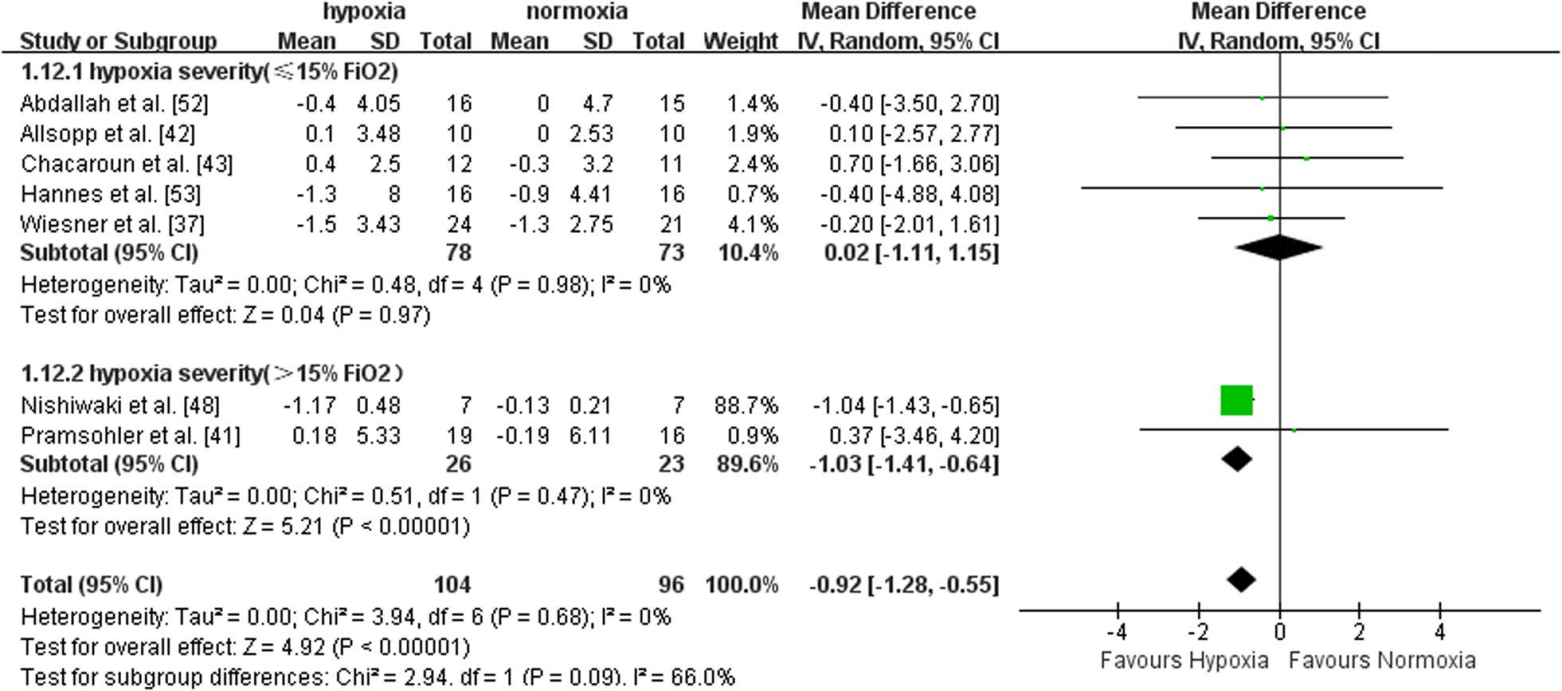
Hypoxia exposure severity subgroup analysis (BMI). Filled green square represents study-specific estimates, and filled diamond represents pooled estimates of random-effects. *CI* confidence interval, *SD* standard deviation

**Fig. 6:**
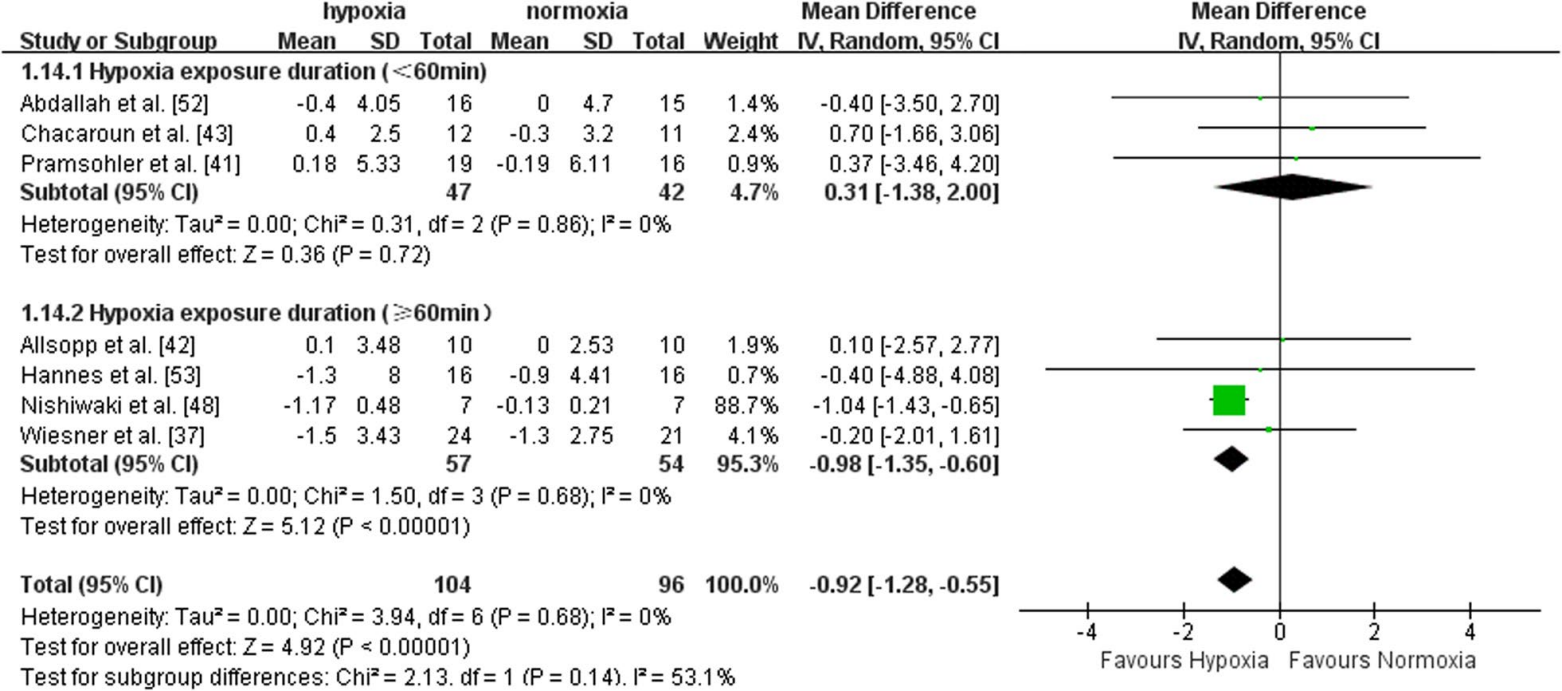
Hypoxia exposure duration (mins per session) subgroup analysis (BMI). Filled green square represents study-specific estimates, and filled diamond represents pooled estimates of random-effects. *CI* confidence interval, *SD* standard deviation

Seven studies recruiting middle-aged (n = 5) and older adults (n = 2) reported the effects of exercise with or without hypoxia on BMI. There was a significant effect in favour of hypoxia on BMI compared to normoxia (MD = -0.92, 95%CI -1.28 to -0.55, I^2^ = 0, p < 0.00001). Subgroup analysis showed a larger effect of exercise in hypoxia *versus* normoxia in middle-aged adults (MD = -0.95, 95%CI -1.32 to -0.58, I^2^ = 0, p < 0.00001), but not in older adults (MD = -0.19, 95%CI -2.00 to 2.38, I^2^ = 0, p = 0.87) (Fig. 4). Compared to normoxia, the effect of exercise in hypoxia for improving BMI was significantly larger with FiO_2_ > 15% (MD = -1.03, 95%CI -1.41 to -0.64, I^2^ = 0, p < 0.00001), but not with FiO_2_ ≤ 15% (MD = 0.02, 95%CI -1.11 to 1.15, I^2^ = 0, p = 0.97) (Fig. 5). Compared with shorter (< 60 min per session) hypoxia exposure duration (MD = 0.31, 95%CI -1.38 to 2.00, I^2^ = 0, p = 0.72), longer hypoxia exposure duration (≥ 60 min per session) induced larger BMI improvement (MD = -0.98, 95%CI -1.35 to -0.60, I^2^ = 0, p < 0.00001) (Fig. 6).

#### Body fat

**Fig. 7:**
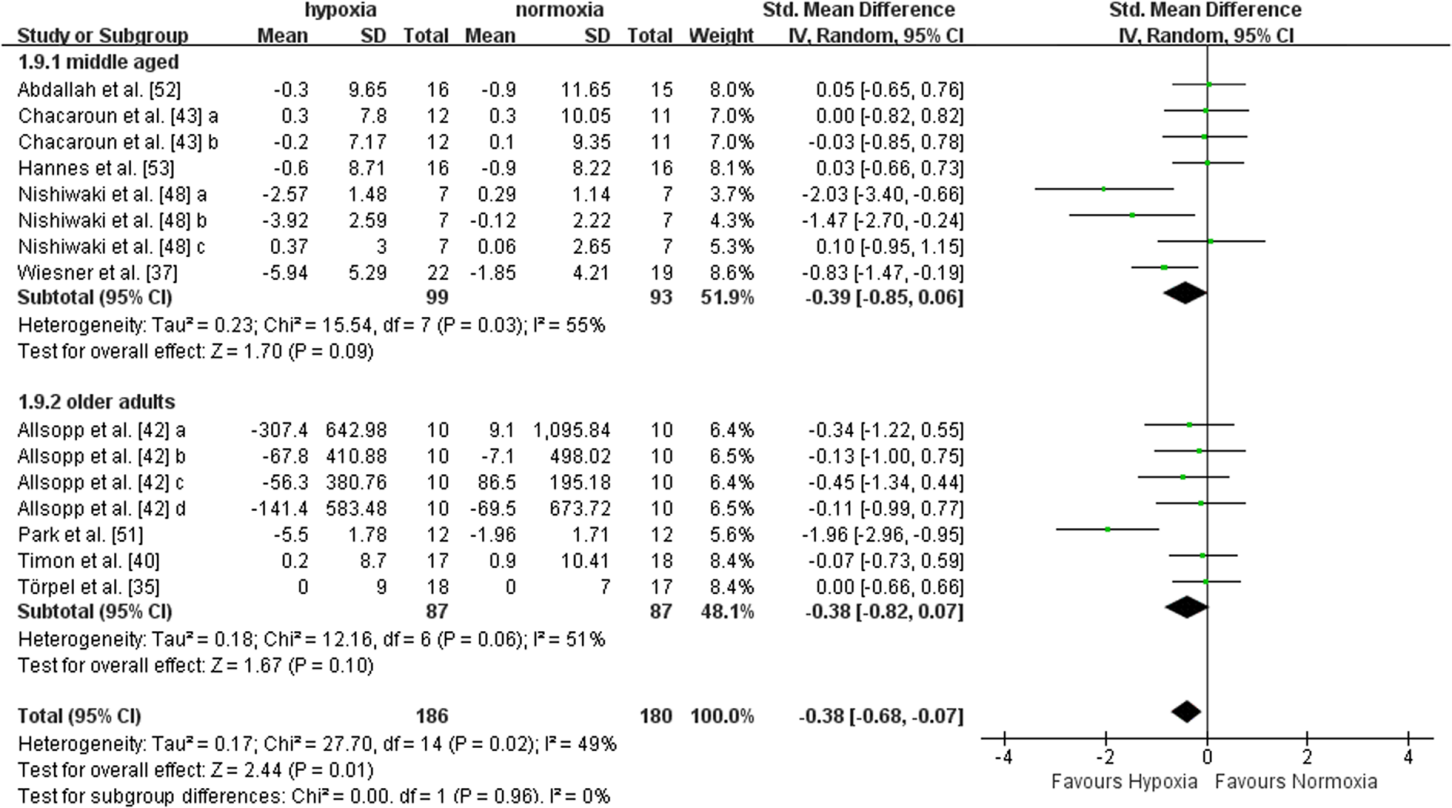
Meta-analysis of the effects of exercise in hypoxia *versus* normoxia on body fat in middle-aged and older adults. ‘a’, ‘b’, ‘c’, ‘d’ represents different outcome indicators of body fat reported in the same study. Filled green square represents study-specific estimates, and filled diamond represents pooled estimates of random-effects. *CI* confidence interval, *SD* standard deviation

**Fig. 8:**
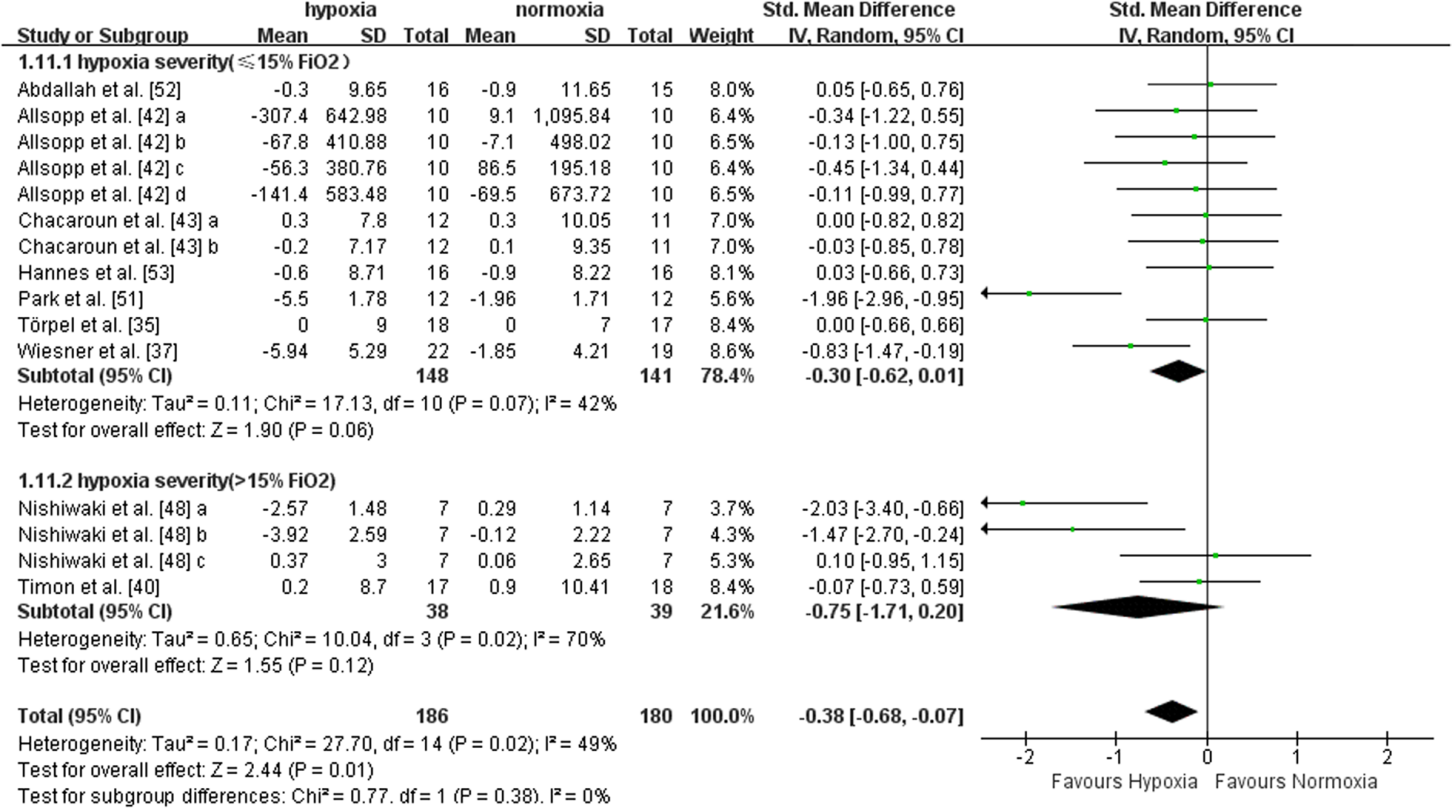
Hypoxia exposure severity subgroup analysis (body fat). ‘a’, ‘b’, ‘c’, ‘d’ represents different outcome indicators of body fat reported in the same study. Filled green square represents study-specific estimates, and filled diamond represents pooled estimates of random-effects. *CI* confidence interval, *SD* standard deviation

**Fig. 9:**
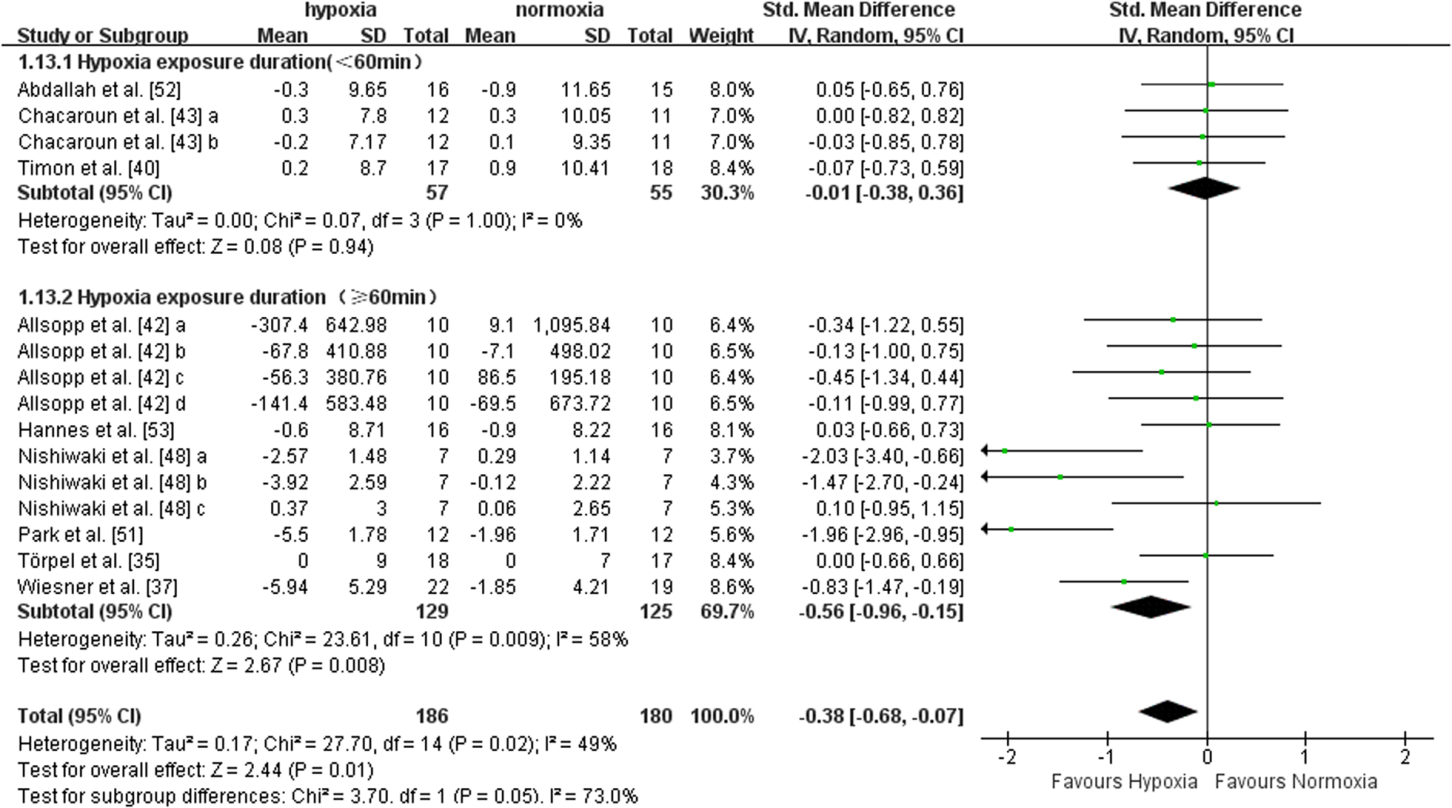
Hypoxia exposure duration (mins per session) subgroup analysis (body fat). ‘a’, ‘b’, ‘c’, ‘d’ represents different outcome indicators of body fat reported in the same study. Filled green square represents study-specific estimates, and filled diamond represents pooled estimates of random-effects. *CI* confidence interval, *SD* standard deviation

Nine studies with a total of 15 data points reported outcome indicators concerning body fat (i.e., fat mass, fat mass percentage, and body fat percentage). Five studies included middle-aged adults, and four studies included older adults. There was a significant effect in favour of hypoxia on body fat compared to normoxia (SMD = -0.38, 95%CI -0.68 to -0.07, I^2^ = 49%, p = 0.01). The effects were not different between middle-aged (SMD = -0.39, 95%CI -0.85 to 0.06, I^2^ = 55%, p = 0.09) and older adults (SMD = -0.38, 95%CI -0.82 to 0.07, I^2^ = 51%, p = 0.10), according to subgroup analysis (Fig. 7). The effect of exercise in hypoxia at FiO_2_ > 15% (SMD = -0.30, 95%CI -0.62 to 0.01, I^2^ = 42%, p = 0.06) was not larger than that at FiO_2_ ≤ 15% (SMD = -0.75, 95%CI -1.71 to 0.20, I^2^ = 70%, p = 0.12) (Fig. 8). Compared with shorter (< 60 min per session) hypoxia exposure duration (SMD = -0.01, 95%CI -0.38 to 0.36, I^2^ = 0%, p = 0.94), longer hypoxia exposure duration (≥ 60 min per session) induced larger reductions in body fat (SMD = -0.56, 95%CI -0.96 to -0.15, I^2^ = 58%, p = 0.008) (Fig. 9).

## Discussion

This meta-analysis is the first to compare the effects of hypoxia conditioning with similar training in normoxia on body composition in middle-aged and older adults. A total of twelve studies with 335 participants training using various exercise modalities (resistance exercise, aerobic exercise, whole-body vibration training, high-intensity interval training, and a combination of aerobic and resistance exercise) were selected. Overall, results showed that hypoxia conditioning induces larger improvements in body fat and BMI, but not in lean mass.

### Effects of hypoxia conditioning on body fat

This meta-analysis demonstrated that, compared to normoxia, hypoxia conditioning is more effective for reducing body fat in adults aged between 40 and 80 years old. Our results for body fat are consistent with previous studies [37, 47, 48, 51] and are also in accordance with findings obtained in cohorts of different age (16 to 40 years old) and individuals with obesity [30, 54, 55]. One potential underlying mechanism to explain reported lower body fat with hypoxia conditioning relates to appetite reduction, eventually through a decrease of acylated ghrelin concentration associated with acute hypoxic exposure, thus restricting energy intake and contributing to energy deficit [56]. Hence, energy deficit is the most important consideration for losing weight, regardless of the dietary approach being used [57]. It was reported that hypoxia could modulate leptin levels, and increase adipocytokines, metabolic rate, mitochondrial function, and fat oxidation [56, 58]. Fat oxidation can be enhanced by an upregulation in the activity of mitochondrial enzymes [59]. On the basis that hypoxic inducible factor-1 expression induced by repeated hypoxic exposure can maximize the number and efficiency of mitochondria, exercise training in oxygen-deprived conditions is now considered a viable therapeutic approach for treating obesity [39, 47].

There were several differences, including intervention design, dietary control, and age span, between the included studies despite the relatively low heterogeneity. Firstly, we found that there was one study [48] with a much larger effect than other investigations for reducing body fat. While hypobaric hypoxia was used in this study, all others included exercise sessions only performed in normobaric hypoxia. This difference in the nature of the hypoxic stimulus may be a source for the heterogeneity observed. Besides, daily dietary and energy intake of participants should be strictly controlled given that it might significantly influence the magnitude of fat loss [57]. None of the included studies reported the daily energy intake during the intervention period, while six studies simply requested (i.e., not verified) participants to maintain their normal diet. As a result, studies investigating the effect of hypoxia conditioning with a strict control of daily energy intake may need to be conducted in order to evaluate the effects of any additional benefits of hypoxia exposure for reducing body fat.

### Effects of hypoxia conditioning on body mass index

Hypoxia conditioning favoured a reduction in BMI compared to normoxia. Baseline BMI levels of tested participants ranged from 23.9–33.1 kg/m^2^, indicating that only overweight-to-obese individuals were recruited. Given that lean mass did not increase significantly in most of the included studies (see next section), and the overall effect calculated from this meta-analysis was not significant either, we could speculate that BMI improvements are mainly due to a reduction in body fat. Among all investigations, the study by Nishiwaki et al. [48], is the only one showing a significant effect on BMI. Interestingly, the exercise program was conducted in hypobaric hypoxia (simulated altitude of 2000 m or ~ FiO_2_ = 16.4%), while training sessions in all the other included studies were performed in normobaric hypoxia. Higher metabolic rate, reduced food intake, and a rise in leptin levels caused by hypobaric hypoxia [58] could explain why the largest effects on both BMI and body fat were found in the only hypobaric hypoxia study [48]. Regardless, the validity of BMI as a body fat predictor continues to be debated since individuals with high muscle mass may be incorrectly classified as obese based on their BMI [60]. However, participants included in this meta-analysis were inactive middle-aged and older adults with normal muscle mass. Therefore, larger improvements in BMI using hypoxic exposure should be considered a positive outcome of managing body composition.

### Effects of hypoxia conditioning on lean mass

Hypoxia conditioning was not superior than equivalent normoxic training to modify lean mass, while there were also no significant differences between middle-aged and older adults. These findings are inconsistent with previous observations made in younger adults [61–64]. These authors reported that resistance exercise at moderate intensity (70–85% 1RM) in hypoxia is more effective for improving lean body mass and fibre cross-sectional area than higher exercise intensities (> 85%1RM) in normoxia. The metabolic stress caused by both resistance exercise and hypoxia can lead to an increased muscle fibre recruitment, elevated hormonal release, altered myokine production, and cellular swelling [62, 65], in turn explaining larger increases in force production capacity compared to normoxic interventions. In our meta-analysis, however, the superiority of hypoxia conditioning compared to normoxia to boost muscle mass in middle-aged and older adults could not be verified.

Several suggestions can be made to explain why hypoxic exposure had no additional benefits on muscle mass. First, the hormonal response observed in younger adults might not occur, or occur at a relatively low degree, in middle-aged and older adults. In fact, resistance training in hypoxia suppressed the growth hormone response to exercise in older adults, while other hormones and metabolic markers were unaffected both acutely and chronically by hypoxia [66]. Reportedly, acute erythropoietin expression after hypoxia exposure in young people was significantly higher than in older individuals [67]. Secondly, exercise interventions reviewed here may not be optimally designed for muscle growth in middle-aged and older adults. Of all studies that considered lean mass as a main outcome, three of them used resistance exercise, four selected aerobic training, two performed whole-body vibration training, one used high-intensity interval training, and one combined aerobic and resistance exercises. A progressive overload is an effective strategy for inducing muscle hypertrophy using resistance exercise, but not necessarily for other forms of exercise [68, 69]. Consequently, additional hypoxia exposure might not be effective to induce muscle hypertrophy for ‘non-resistance’ exercise modalities. Thirdly, given that sufficient energy and protein intake are required to maximize the hypertrophic response, the apparent lack of significant effect in the included resistance training studies may be partly due to an uncontrolled diet [70]. Pending confirmatory research, a hypoxia-induced appetite reduction may also explain this observation. Future studies on resistance exercise in hypoxia should consider interventions designed to specifically induce a hypertrophic response (e.g., intensity, volume, time under tension, Rating of Perceived Exertion (RPE)) [65], with a strict control of energy and protein intake.

### Subgroup analysis

#### Age

Subgroup analysis showed that the effects of exercise in hypoxia on body fat did not differ between middle-aged and older adults. In normal weight and active subjects, ageing had no deleterious effect on cardiac and ventilatory responses to hypoxia [71]. As such, middle-aged and older adults likely responding similarly to exercise in hypoxia should achieve quite comparable benefits with hypoxia conditioning. Improved insulin sensitivity through hypoxic training is linked with a decrease in body fat in both obese and elderly individuals [47]. However, several factors could contribute to age-induced modifications of body composition, such as decreases in physical activity, energy production within the cell, and mitochondrial protein synthesis. The exact mechanisms responsible for comparable effects of exercise in either hypoxia or normoxia for reducing body fat remain unknown.

#### Hypoxia severity and duration

Hypoxia severity ranged from 2000 to 4500 m simulated altitudes (FiO_2_ = 12.0–16.4%) in included studies. In our subgroup analysis, we used an arbitrary value of FiO_2_ = 15% to delimit moderate hypoxia (FiO_2_ > 15%) and more severe (FiO_2_ ≤ 15%) hypoxic levels [47]. A previous meta-analysis showed significant body weight and fat mass reductions induced by moderate altitude exposure combined with exercise [72]. Contrastingly, in our study, there was no significant effect on improving body fat that favoured hypoxia conditioning for groups exposed to either moderate or more severe hypoxia. However, considering body fat, a moderate effect size favouring hypoxia was obtained in the subgroup with less severe hypoxia levels (FiO_2_ > 15%). This observation may be due to the limited number of eligible studies. Besides, hypoxia exposure duration per session varied among studies that used moderate hypoxia, which may explain why non-significant effects were reported. Within the moderate hypoxia subgroup, one study exposed participants to hypoxia for 120 min per session (with significantly larger effects) [48], while the other one only used 45 min (with smaller effect) [43]. Compared with normoxia, the effects of hypoxia conditioning on both body fat (moderate) and BMI (large) were significant in the subgroup with longer exposure duration (≥ 60 min). Studies reported that sufficient daily hypoxia exposure time and prolonged duration of the training program significantly reduced body fat and body weight [72, 73], which is in agreement with our findings. Therefore, exercise in moderate hypoxia combined with exposure duration of at least 60 min is recommended for improving body fat and BMI in middle-aged and older adults.

### Limitations

This study is not without limitations. With most of the included studies published in the past five years, hypoxia conditioning can be considered a fast-developing research area. Because only a few studies focused on the effects of exercise in hypoxia on body composition in middle-aged and older adults, the number of eligible studies was small. The heterogeneity of the protocols used, including resistance exercise, aerobic exercise, whole-body vibration training, high-intensity interval training, and a combination of aerobic and resistance exercise, poses a challenge when determining the effects of hypoxic conditioning. This challenge is further exacerbated by the limited number of investigations available for subgroup analysis. As such, we were unable to conduct subgroup analysis of the effects of different exercise modes, intensities, volumes and frequencies, all known to modulate cardio-metabolic stimulation and eventually the efficacy of hypoxia conditioning.

The inclusion of studies on endurance exercise, which is not anticipated to increase lean mass regardless of oxygen conditions, in the meta-analysis focusing on changes in lean mass may introduce inaccuracies. Even within a given type of exercise modality, such as resistance exercise or high-intensity interval training, the effect of adding hypoxia is likely to differ depending on a combination of factors. These factors include exercise structure (i.e., exercise-to-rest ratio, exercise duration), the hypoxic dose (i.e., exposure duration and severity), and the background of the participants being tested [74]. It is common to observe variable results, with some individuals experiencing greater improvements in health markers with a specific form of training, a higher training dose, and/or hypoxia [75].

Another limitation was the utilization of an age limit of > 40 years to select studies that focused on middle-aged or older individuals. This criterion disregards the mean age of the population under investigation in each study, potentially resulting in the inclusion of individuals below 40 years old and introducing a potential source of bias. This is especially significant considering the SD reported for age by some studies (Table 1).

### Further research considerations

In the absence of a well-accepted metric for defining the ‘hypoxic dose’ (severity and duration of the hypoxic stimulus), it remains challenging to directly compare literature findings. Recently, an index that integrates both the external (FiO_2_) and internal (arterial oxygen saturation or SpO_2_) stimuli to characterize individual responses to normobaric hypoxia has been introduced (i.e., the so-called ‘SpO_2_ to FiO_2_ ratio’ [74]). This metric based upon the magnitude of the stimulus (i.e., SpO_2_ as a reflection of the ‘internal’ physiological stimulation), as opposed to the altitude elevation (i.e., only representing the ‘external’ stress), and also considering the duration of hypoxia exposure might be relevant for comparing studies [76]. Additionally, sufficient energy and protein intake are required to maximize the hypertrophic response in terms of the effects of an intervention on lean mass. However, none of the studies included in this meta-analysis were designed to specifically induce a maximal hypertrophic response (e.g., intensity, volume, time under tension, RPE), with a strict control of energy and protein intake. In addition to modulating energy intake, exposure to hypoxia likely increases the body’s reliance on carbohydrate as a fuel for substrate oxidation in reference to normoxia [77]. While these considerations are important when designing interventions to lose weight and effectively manage body composition, diet was apparently not carefully controlled in most included studies. Future studies are required to delineate the isolated and combined effects of hypoxia and diet. Finally, the included studies did not account for potential differences between men and women, and it is plausible that a sex-related effect could have influenced the observed results.

## Conclusion

Hypoxia conditioning, compared to equivalent training in normoxia, induced greater body composition improvement in terms of body fat and BMI in middle-aged and older adults. Adding hypoxia exposure to exercise interventions is a viable therapeutic solution to effectively manage body composition in ageing population. Our findings provide a valuable starting point for health professionals to explore innovative treatment options aimed at improving body composition in middle-aged and older adults. It is important to note that when prescribing hypoxic conditioning, there are multiple effective approaches, and no single modality can be universally recommended as the best for improving health outcomes. However, a guiding principle is to incorporate hypoxia conditioning sessions lasting at least 60 min within a moderate hypoxic environment (FiO_2_ > 15%) to support middle-aged and older adults in achieving improved health outcomes. It is crucial to consider contextual factors such as individual characteristics, exercise preferences, availability of time and resources, as well as access to hypoxicators and/or simulated altitude chambers when implementing these interventions.

## Data Availability

The datasets used and analysed during the current study are available from the corresponding author on reasonable request.

## Abbreviations

BMI: Body Mass Index
CI: Confidence Intervals
CNKI: China National Knowledge Infrastructure
FiO_2_: Fraction of Inspired Oxygen
MD: Mean Difference
RPE: Rating of Perceived Exertion
SD: Standard Deviation
SE: Standard Error
SEM: Standard Error of Mean
SMD: Standardized Mean Difference
SpO_2_: Arterial Oxygen Saturation
